# Surgical outcomes of gastroenterological surgery in Japan: Report of the National Clinical Database 2011‐2017

**DOI:** 10.1002/ags3.12258

**Published:** 2019-05-20

**Authors:** Hiroshi Hasegawa, Arata Takahashi, Yoshihiro Kakeji, Hideki Ueno, Susumu Eguchi, Itaru Endo, Akira Sasaki, Shuji Takiguchi, Hiroya Takeuchi, Masaji Hashimoto, Akihiko Horiguchi, Tadahiko Masaki, Shigeru Marubashi, Kazuhiro Yoshida, Hiroyuki Konno, Mitsukazu Gotoh, Hiroaki Miyata, Yasuyuki Seto

**Affiliations:** ^1^ The Japanese Society of Gastroenterological Surgery Tokyo Japan; ^2^ Department of Health Policy and Management School of Medicine Keio University Tokyo Japan; ^3^ Department of Healthcare Quality Assessment Graduate School of Medicine The University of Tokyo Tokyo Japan

**Keywords:** gastroenterological surgery, National Clinical Database, surgical outcome

## Abstract

**Background:**

The Japanese National Clinical Database (NCD) is a large‐scale, nationwide, web‐based data entry system that is linked to the surgical board certification system and covers almost all surgical cases carried out in Japan.

**Aim:**

To evaluate outcomes according to the gastroenterological section of the NCD.

**Methods:**

The 115 surgical procedures stipulated by the “Training Curriculum for Board‐Certified Surgeons in Gastroenterology” were registered from 2011 to 2017. The number of surgeries, preoperative comorbidities, and short‐term outcomes were compared between registration periods.

**Results:**

In total, 3 818 414 cases have been registered. More than 70% of all surgeries were carried out at certified institutions. The annual number of cases has been increasing year after year, and the aged population has also been increasing. Although the rates of preoperative comorbidities and postoperative complications have been increasing, the postoperative mortality rate has remained relatively low; in 2017, the 30‐day mortality rate was 1.0% among those who underwent esophagectomy, 0.7% among those who underwent distal gastrectomy, 1.1% among those who underwent total gastrectomy, 1.3% among those who underwent right hemicolectomy, 0.5% among those who underwent low anterior resection, 1.3% among those who underwent hepatectomy, and 1.3% among those who underwent pancreaticoduodenectomy. The annual rate of endoscopic surgery dramatically increased over 7 years between 2011 and 2017, especially for low anterior resection (29.5%‐62.6%) and esophagectomy (31.0%‐56.1%).

**Conclusion:**

This database is expected to ensure the quality of the board‐certification system and surgical outcomes in gastroenterological surgery.

## 1 INTRODUCTION

The Japanese National Clinical Database (NCD) is a large‐scale, nationwide, web‐based data entry system linked to the surgical board certification system, and covers almost all surgical cases (90%‐95%) carried out in Japan.^1^ The NCD was created in April 2010 with major support from the Japan Surgical Society and the Japanese Society of Gastroenterological Surgery (JSGS).^2^ Fifteen professional societies joined the NCD in 2018. The NCD has collected data on more than 9 690 000 surgical cases from almost 5000 facilities from January 2011 to December 2017.

In the gastroenterological section of the NCD, the JSGS selected 115 gastrointestinal operative procedures as important for the board certification system and eight main procedures (ie, esophagectomy, distal gastrectomy, total gastrectomy, right hemicolectomy, low anterior resection, hepatectomy, pancreaticoduodenectomy, and surgery for acute diffuse peritonitis) as especially important in terms of medical standards for improvement of surgical quality. All surgical cases are registered in the NCD with input of postoperative complications for the 115 procedures, and with detailed input such as comorbidities and morbidities for the eight main procedures.^3^ Risk models of mortality for the eight main procedures were established using approximately 120 000 surgical cases registered in 2011.^4^, ^5^, ^6^, ^7^, ^8^, ^9^, ^10^, ^11^ Risk models of morbidity for the eight main procedures were also established using approximately 250 000 surgical cases registered in 2011 and 2012.^12^, ^13^, ^14^, ^15^, ^16^, ^17^ Using the risk models of morbidity and mortality, the risk calculator was created. The risk calculator adjusts the risks of patients, provides the predicted morbidity and mortality of patients after inputting the preoperative data, and has been available on the websites of the participating hospitals since 2015.^2^ To evaluate the reliability of data collection, the JSGS started data verification activity in 2016 and found high accuracy of data entry.^18^

Following up on the 2011‐2016 Report,^3^ we herein summarize the 2011‐2017 data in the NCD based on gastroenterological surgery information on 3 818 414 cases of surgeries carried out and recorded from 2011 to 2017 along with the data on perioperative complications.

## 2 SUBJECTS AND METHODS

Methods were the same as previously reported.^3^ Subjects were patients whose surgical data were recorded in the NCD, and who underwent one or more of the 115 surgical procedures stipulated by the “Training Curriculum for Board‐Certified Surgeons in Gastroenterology,” using the “New classification of surgical difficulty.” The board certification system of the JSGS consists of board‐certified training institutions and board‐certified surgeons in gastroenterological surgery.^19^ One requirement for board‐certified training institutions is having carried out 600 or more gastroenterological operations as determined by the certifying committee (of which more than 120 gastroenterological operations were essential major surgery) in the last 3 years. Board‐certified surgeons are required to have received gastroenterological surgical training for more than 5 years according to the training curriculum in a board‐certified training institution authorized by the JSGS and to have carried out 450 or more gastroenterological operations. We targeted data from 2011 to 2017, adding the data of complications to cases that have already been reported in the 2011‐2016 Report on the 115 gastroenterological surgical procedures. Complications included surgical site infection (SSI), wound dehiscence, anastomotic leakage, pancreatic fistula, bile leakage, pneumonia, unplanned intubation, pulmonary embolism, ventilator‐assisted respiration longer than 48 hours, progressive renal insufficiency, acute renal failure, urinary tract infection, cerebrovascular accident with neurological deficit, coma longer than 24 hours, peripheral nerve injury, cardiac arrest requiring cardiopulmonary resuscitation, myocardial infarction, bleeding complication defined as transfusion in excess of one unit of blood, deep venous thrombosis, and sepsis. Postoperative complications were categorized into six grades according to the Clavien‐Dindo (C‐D) classification.^20^ In this study, complications of grade III (complications requiring intervention) or higher were defined as severe complications. Furthermore, among the 115 surgical procedures, we separated and studied the eight main operative methods that we deemed important in terms of medical standards.

We clarified the number of surgical cases and the mortality rates related to the 115 selected gastroenterological operative procedures. We also clarified the changes over time in the annual number of surgical cases, preoperative morbidity rates, and mortality rates related to the eight main operative procedures from 2011 to 2017. We also comparatively studied patient gender, age groups, institution type, and percentage of surgeries carried out by certified surgeons related to the eight main operative procedures.

The following points need to be considered in the interpretation of the data reported here. (i) As a maximum of eight operative procedures can be recorded for each case in the NCD, the total number of surgeries in “Results of the 115 gastroenterological surgical procedures for board certification system” is not the actual total number of surgical cases; (ii) cases with abnormal data or missing information in patient age, gender, or 30‐day postoperative status were excluded; (iii) cases in which several operative methods were carried out simultaneously were tallied for all operative methods; (iv) postoperative 30‐day mortality included all cases of mortality within 30 days after surgery regardless of pre‐ or post‐discharge status. Calculation of operative mortality included all patients who died during the index hospitalization, including hospital stays of up to 90 days, and any patient who died after hospital discharge within 30 days of the operative date.

## 3 RESULTS

### 3.1 Analysis of cases who underwent one or more of the 115 selected gastrointestinal operative procedures in the “Training Curriculum for Board‐Certified Surgeons in Gastroenterology”

The total number of cases that underwent one or more of the 115 selected gastroenterological surgical procedures reported in the NCD between January 1, 2011 and December 31, 2017 was 3 818 414. Based on organ involvement, 61 242 cases involved the esophagus (1.6%); 507 827 cases the stomach and duodenum (13.3%); 1 409 527 cases the small intestine and colon (36.9%); 360 101 cases the rectum and anus (9.4%); 182 462 cases the liver (4.8%); 894 793 cases the gallbladder (23.4%); 117 503 cases the pancreas (3.1%); 26 135 cases the spleen (0.7%), and 258 824 cases other organs (6.8%). The annual number of surgical cases for each organ generally showed an increasing trend over time except for surgeries on the stomach and duodenum, and surgeries on the spleen. The male : female ratio was approximately 8:2 for surgeries on the esophagus, 7:3 for surgeries on the stomach and duodenum, 7:3 for surgeries on the liver, and 6:4 for surgeries on other organs. Year by year, the percentages of older patients have been increasing for all organs (Table 1).

**Table 1:** Annual changes in percentage of surgeries by gender and age group for 115 selected GI operative procedures in the training curriculum for board‐certified surgeons in gastroenterology classified according to target organ

Organ	Year	No. of surgeries	Percentage by gender		Percentage according to age group (y)					
			Male	Female	<60	60 to <65	65 to <70	70 to <75	75 to <80	≥80
Esophagus	2011	7246	81.8	18.2	22.5	19.6	21.1	18.7	12.0	6.0
	2012	8819	82.2	17.8	22.1	19.7	20.0	19.5	12.9	6.0
	2013	8642	81.5	18.5	20.8	17.5	21.0	20.6	13.2	6.9
	2014	9021	81.5	18.4	20.8	16.5	21.4	20.9	13.8	6.6
	2015	8943	80.8	19.2	19.6	15.3	22.4	22.5	13.1	7.1
	2016	9212	79.6	20.4	20.1	14.4	22.9	20.5	14.5	7.5
	2017	9359	80.0	20.0	19.3	13.4	24.4	19.4	15.5	8.0
Stomach and duodenum	2011	66 740	68.0	32.0	20.1	14.4	14.0	17.1	16.4	18.0
	2012	76 186	68.3	31.7	18.9	14.4	14.5	17.1	16.4	18.6
	2013	75 583	67.9	32.1	18.6	13.1	15.5	17.2	16.9	18.7
	2014	74 920	67.6	32.4	17.9	12.1	16.0	17.8	16.7	19.5
	2015	73 877	67.8	32.2	17.4	11.1	17.1	17.8	16.6	19.9
	2016	72 234	67.8	32.2	17.0	10.2	18.1	17.1	16.6	21.0
	2017	68 287	67.2	32.8	16.3	9.9	17.5	17.3	17.2	21.8
Small intestine and colon	2011	151 143	56.7	43.3	37.4	10.9	10.5	12.1	12.2	16.9
	2012	184 810	56.7	43.3	36.4	10.7	10.7	12.2	12.5	17.4
	2013	198 677	56.9	43.1	35.6	10.1	11.3	12.7	12.4	17.8
	2014	206 857	56.9	43.1	34.7	9.4	12.0	13.1	12.4	18.4
	2015	214 453	57.1	42.9	34.0	8.9	12.9	13.1	12.3	18.7
	2016	218 228	57.3	42.7	33.7	8.4	13.6	12.5	12.4	19.3
	2017	235 359	56.7	43.3	32.7	8.0	13.2	12.7	12.9	20.5
Rectum and anus	2011	41 061	59.1	40.9	22.0	16.1	14.6	15.4	14.2	17.7
	2012	49 704	58.3	41.7	22.3	14.8	14.6	15.5	14.3	18.5
	2013	49 980	58.0	42.0	20.9	13.9	15.2	16.1	14.6	19.3
	2014	51 454	58.3	41.7	20.4	13.1	16.0	16.4	14.2	19.9
	2015	56 092	57.8	42.2	22.3	11.8	16.7	15.7	14.0	19.4
	2016	55 666	57.3	42.7	22.0	11.1	17.9	15.0	13.6	20.4
	2017	56 144	56.7	43.3	22.2	10.2	17.3	15.1	14.2	21.0
Liver	2011	22 855	67.3	32.7	22.2	16.5	16.3	18.7	17.2	9.2
	2012	26 288	66.3	33.7	22.1	15.7	16.7	18.0	17.4	10.2
	2013	25 814	66.1	33.9	21.3	14.6	17.6	18.7	17.3	10.5
	2014	26 518	66.3	33.7	21.5	13.7	18.1	19.8	16.6	10.3
	2015	26 378	65.7	34.3	20.8	12.8	18.9	19.4	16.5	11.5
	2016	27 212	66.4	33.6	20.3	11.5	20.5	18.6	17.0	12.1
	2017	27 397	65.8	34.2	20.1	11.0	20.2	18.8	17.2	12.7
Gallbladder	2011	103 183	54.5	45.4	34.3	14.0	12.2	13.8	12.8	13.0
	2012	122 513	55.2	44.8	32.9	13.8	12.4	13.9	13.2	13.8
	2013	129 162	55.3	44.7	32.6	12.9	13.0	14.2	13.2	14.0
	2014	131 182	55.6	44.4	32.1	11.8	13.9	14.5	13.2	14.5
	2015	133 126	55.6	44.4	32.0	11.2	15.0	14.1	13.0	14.8
	2016	137 360	55.4	44.6	32.6	10.6	15.5	13.1	12.9	15.3
	2017	138 267	55.6	44.4	32.2	10.2	15.1	13.5	13.2	15.8
Pancreas	2011	13 477	59.9	40.1	20.0	15.6	16.9	19.7	17.7	10.2
	2012	15 550	60.0	40.0	19.8	15.2	17.0	19.5	18.2	10.3
	2013	16 380	59.7	40.3	19.1	13.6	18.0	20.7	17.7	10.9
	2014	17 313	59.5	40.5	18.4	12.4	19.0	21.0	18.2	11.1
	2015	17 407	59.1	40.9	18.2	11.3	19.4	21.6	18.1	11.4
	2016	18 238	58.9	41.1	18.2	10.4	19.9	20.4	19.0	12.2
	2017	19 138	59.2	40.8	17.7	9.9	19.5	19.9	20.1	12.9
Spleen	2011	3609	61.3	38.7	35.3	15.6	14.7	14.8	11.9	7.8
	2012	4142	61.4	38.6	32.9	16.3	15.0	15.1	12.9	7.8
	2013	4509	61.8	38.2	30.8	14.9	15.9	16.5	13.1	8.7
	2014	4272	61.8	38.2	29.9	13.0	17.3	17.0	13.8	9.1
	2015	3568	60.4	39.6	29.7	11.4	17.3	16.6	14.1	10.8
	2016	3171	57.3	42.7	31.9	11.7	17.7	15.7	12.5	10.5
	2017	2864	58.7	41.3	31.6	11.0	18.1	16.0	13.3	10.0
Other	2011	23 218	55.0	45.0	32.0	11.9	11.3	13.3	13.8	17.6
	2012	28 779	55.4	44.6	31.1	11.7	11.7	13.8	13.7	18.0
	2013	36 363	53.1	46.9	28.3	10.9	12.7	14.1	14.8	19.1
	2014	39 854	53.7	46.3	28.1	10.1	13.1	14.5	14.4	19.8
	2015	41 465	53.2	46.8	27.4	9.4	14.0	14.5	14.2	20.6
	2016	43 523	54.0	46.0	27.5	9.2	14.6	13.5	14.0	21.2
	2017	45 622	54.1	45.9	27.0	8.2	14.7	13.5	14.6	21.9

Abbreviation: GI, gastrointestinal.

**Figure 1:**
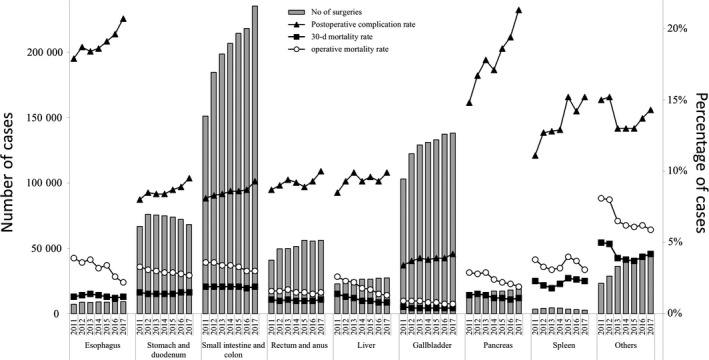
Annual changes in the number of surgeries, postoperative complication rate, operative mortality rate, and 30‐d postoperative mortality rate: analysis of the 115 selected surgical procedures that were classified according to the involved organ. Postoperative complication rate: the rate of complications with Clavien‐Dindo (C‐D) classification of grade III (complications requiring intervention) or higher

In terms of the type of institution in which the surgeries were carried out, more than 70% of all surgeries were carried out at certified institutions, and the percentage of surgeries carried out at certified institutions was particularly high in 2017 for surgeries on the esophagus (92.7%) and pancreas (90.4%). The percentage of surgeries with participation of an anesthesiologist was more than 90% for almost all organs, except 84.8% for the rectum and anus. More than 70% of surgeries on most organs were carried out with the participation of a board‐certified surgeon. In 2017, the percentage of surgeries in which a certified surgeon was the operator was high for surgeries on the esophagus (71.8%), liver (62.5%), and pancreas (63.9%; Table 2). Postoperative complications, operative mortality rates, and 30‐day postoperative mortality rates are shown in Table 3. Complication rates were comparatively higher in 2017 for surgeries on the esophagus (20.7%) and the pancreas (21.3%); however, the mortality rates for procedures on these organs were not so high. Figure 1 shows the number of surgeries, rates of complications and mortality rates among cases who underwent the 115 gastroenterological surgical procedures according to organ involvement. Tables 4, 5, 6, 7, 8, 9, 10, 11, 12 show the number of surgeries carried out using each of the 115 gastroenterological surgical procedures, according to recording year and organ.

**Table 2:** Institution type, anesthesiologist and specialist participation rates in the 115 selected GI operative procedures that were classified according to target organ

Organ	Year	No. of surgeries	Percentage by institution type			Anesthesiologist participation (%)	Board‐certified surgeon participation (%)	Medical practitioners (%)	
			Certified institution	Related institution	Other			Board‐certified surgeons	Non‐board‐certified surgeons
Esophagus	2011	7246	93.5	5.9	0.6	97.0	87.0	62.8	37.2
	2012	8819	78.1	5.9	16.0	97.4	87.0	62.7	37.3
	2013	8642	90.6	7.1	2.4	97.3	88.4	64.4	35.6
	2014	9021	91.1	6.1	2.8	97.9	90.1	67.6	32.4
	2015	8943	91.5	6.0	2.5	97.9	91.1	69.4	30.6
	2016	9212	92.4	5.0	2.6	98.2	91.2	70.0	30.0
	2017	9359	92.7	4.0	3.3	97.9	92.5	71.8	28.2
Stomach and duodenum	2011	66 740	80.2	17.3	2.6	92.8	69.3	35.1	64.9
	2012	76 186	63.5	15.6	20.9	93.5	70.3	35.6	64.4
	2013	75 583	76.3	19.3	4.4	93.3	73.5	37.7	62.3
	2014	74 920	77.0	18.2	4.8	93.6	75.9	39.2	60.8
	2015	73 877	77.1	18.3	4.6	93.9	76.1	39.2	60.8
	2016	72 234	79.6	16.1	4.3	94.6	78.7	41.0	59.0
	2017	68 287	79.6	15.3	5.1	94.8	79.7	41.8	58.2
Small intestine and colon	2011	151 143	76.8	20.2	2.9	88.1	59.2	25.1	74.9
	2012	184 810	60.6	18.2	21.2	88.9	59.9	25.4	74.6
	2013	198 677	72.6	22.2	5.2	89.6	62.7	26.6	73.4
	2014	206 857	73.0	21.4	5.6	90.8	65.4	28.1	71.9
	2015	214 453	73.8	20.7	5.5	91.6	66.3	28.5	71.5
	2016	218 228	75.6	19.0	5.5	92.4	68.1	29.5	70.5
	2017	235 359	76.0	18.0	6.0	92.9	70.1	31.1	68.9
Rectum and anus	2011	41 061	76.9	19.0	4.1	86.3	68.3	36.9	63.1
	2012	49 704	60.4	18.2	21.4	85.7	68.6	37.6	62.4
	2013	49 980	72.9	21.7	5.4	87.3	71.2	39.4	60.6
	2014	51 454	73.5	20.9	5.6	87.9	73.7	41.6	58.4
	2015	56 092	72.5	20.8	6.7	84.9	73.5	41.5	58.5
	2016	55 666	74.1	19.4	6.6	85.7	74.7	42.1	57.9
	2017	56 144	73.8	18.2	8.0	84.8	76.1	43.9	56.1
Liver	2011	22 855	89.3	9.7	1.1	95.6	85.2	55.2	44.8
	2012	26 288	74.2	9.2	16.7	95.4	85.7	57.4	42.6
	2013	25 814	86.3	10.7	2.9	96.3	87.5	57.1	42.9
	2014	26 518	86.3	10.0	3.7	96.4	89.0	59.6	40.4
	2015	26 378	87.3	9.5	3.2	96.6	89.1	59.1	40.9
	2016	27 212	88.4	8.8	2.9	96.8	90.0	59.6	40.4
	2017	27 397	89.0	7.8	3.1	97.1	91.8	62.5	37.5
Gallbladder	2011	103 183	73.9	22.5	3.6	91.8	61.9	26.4	73.6
	2012	122 513	57.5	19.6	22.9	92.1	62.8	26.3	73.7
	2013	129 162	69.9	24.1	5.9	92.2	65.4	27.3	72.7
	2014	131 182	70.3	23.3	6.4	92.3	67.4	28.1	71.9
	2015	133 126	70.8	22.8	6.4	92.9	68.4	28.1	71.9
	2016	137 360	72.4	21.3	6.3	93.5	69.4	28.9	71.1
	2017	138 267	72.6	20.1	7.3	93.7	71.4	29.9	70.1
Pancreas	2011	13 477	88.1	10.8	1.2	95.8	85.2	57.7	42.3
	2012	15 550	72.8	8.7	18.5	96.3	86.5	59.9	40.1
	2013	16 380	86.5	11.0	2.4	95.9	87.6	60.2	39.8
	2014	17 313	86.9	9.9	3.3	96.2	89.1	61.3	38.7
	2015	17 407	88.4	9.1	2.4	96.4	90.3	61.6	38.4
	2016	18 238	89.8	8.0	2.3	96.8	91.1	62.4	37.6
	2017	19 138	90.4	7.1	2.5	97.2	92.3	63.9	36.1
Spleen	2011	3609	87.0	11.6	1.4	94.6	75.2	44.9	55.1
	2012	4142	70.5	9.5	20.0	81.7	75.8	44.4	55.6
	2013	4509	83.2	13.8	3.0	95.2	75.4	43.3	56.7
	2014	4272	85.4	11.5	3.1	94.6	77.5	45.2	54.8
	2015	3568	85.6	12.3	2.1	94.8	78.9	45.5	54.5
	2016	3171	86.8	10.1	3.1	95.7	80.5	48.0	52.0
	2017	2864	87.4	9.3	3.3	95.3	82.3	49.1	50.9
Other	2011	23 218	80.2	17.0	2.8	90.3	60.4	27.2	72.8
	2012	28 779	65.7	15.2	19.1	91.0	61.1	27.6	72.4
	2013	36 363	76.1	19.3	4.6	91.5	63.4	28.5	71.5
	2014	39 854	76.6	18.2	5.1	91.9	64.9	29.7	70.3
	2015	41 465	78.0	17.2	4.8	92.4	65.6	29.4	70.6
	2016	43 523	79.4	15.8	4.8	92.7	67.3	30.3	69.7
	2017	45 622	80.1	14.8	5.1	93.1	69.7	32.3	67.7

Abbreviation: GI, gastrointestinal.

**Table 3:** No. of surgeries, postoperative complication rates and mortality rates in the 115 selected GI operative procedures that were classified according to target organ

Organ	Year	No. of surgeries	No. of postoperative complications^a^/rate (%)	No. of postoperative 30‐d mortalities/rate (%)	No. of postoperative 90‐d mortalities/rate (%)
Esophagus	2011	7246	1294/17.9	87/1.2	279/3.9
	2012	8819	1653/18.7	117/1.3	315/3.6
	2013	8642	1593/18.4	121/1.4	327/3.8
	2014	9021	1679/18.6	115/1.3	289/3.2
	2015	8943	1709/19.1	103/1.2	304/3.4
	2016	9212	1805/19.6	100/1.1	238/2.6
	2017	9359	1938/20.7	108/1.2	208/2.2
Stomach and duodenum	2011	66 740	5354/8.0	992/1.5	2183/3.3
	2012	76 186	6447/8.5	1085/1.4	2381/3.1
	2013	75 583	6380/8.4	1059/1.4	2269/3.0
	2014	74 920	6328/8.4	1064/1.4	2174/2.9
	2015	73 877	6418/8.7	1007/1.4	2110/2.9
	2016	72 234	6413/8.9	1066/1.5	2016/2.8
	2017	68 287	6455/9.5	1046/1.5	1863/2.7
Small intestine and colon	2011	151 143	12 184/8.1	2943/1.9	5390/3.6
	2012	184 810	15 395/8.3	3564/1.9	6583/3.6
	2013	198 677	16 709/8.4	3723/1.9	6803/3.4
	2014	206 857	17 776/8.6	3822/1.9	6961/3.4
	2015	214 453	18 372/8.6	4019/1.9	7092/3.3
	2016	218 228	19 020/8.7	3933/1.8	6621/3.0
	2017	235 359	21 854/9.3	4588/1.9	7118/3.0
Rectum and anus	2011	41 061	3584/8.7	395/1.0	676/1.6
	2012	49 704	4488/9.0	462/0.9	802/1.6
	2013	49 980	4684/9.4	517/1.0	858/1.7
	2014	51 454	4711/9.2	449/0.9	792/1.5
	2015	56 092	4986/8.9	519/0.9	824/1.5
	2016	55 666	5194/9.3	503/0.9	766/1.4
	2017	56 144	5600/10.0	556/1.0	829/1.5
Liver	2011	22 855	1933/8.5	309/1.4	590/2.6
	2012	26 288	2454/9.3	310/1.2	605/2.3
	2013	25 814	2549/9.9	275/1.1	575/2.2
	2014	26 518	2466/9.3	246/0.9	481/1.8
	2015	26 378	2537/9.6	234/0.9	451/1.7
	2016	27 212	2543/9.3	222/0.8	382/1.4
	2017	27 397	2724/9.9	214/0.8	364/1.3
Gallbladder	2011	103 183	3473/3.4	483/0.5	946/0.9
	2012	122 513	4587/3.7	531/0.4	1082/0.9
	2013	129 162	4982/3.9	546/0.4	1130/0.9
	2014	131 182	5020/3.8	569/0.4	1097/0.8
	2015	133 126	5231/3.9	541/0.4	1036/0.8
	2016	137 360	5320/3.9	559/0.4	980/0.7
	2017	138 267	5761/4.2	576/0.4	968/0.7
Pancreas	2011	13 477	1994/14.8	175/1.3	386/2.9
	2012	15 550	2595/16.7	213/1.4	437/2.8
	2013	16 380	2917/17.8	211/1.3	482/2.9
	2014	17 313	2966/17.1	195/1.1	423/2.4
	2015	17 407	3229/18.6	185/1.1	379/2.2
	2016	18 238	3543/19.4	185/1.0	390/2.1
	2017	19 138	4076/21.3	219/1.1	365/1.9
Spleen	2011	3609	400/11.1	83/2.3	137/3.8
	2012	4142	528/12.7	84/2.0	138/3.3
	2013	4509	575/12.8	79/1.8	139/3.1
	2014	4272	549/12.9	88/2.1	137/3.2
	2015	3568	543/15.2	88/2.5	144/4.0
	2016	3171	449/14.2	76/2.4	117/3.7
	2017	2864	434/15.2	65/2.3	89/3.1
Others	2011	23 218	3494/15.0	1163/5.0	1887/8.1
	2012	28 779	4388/15.2	1399/4.9	2293/8.0
	2013	36 363	4712/13.0	1401/3.9	2346/6.5
	2014	39 854	5176/13.0	1521/3.8	2489/6.2
	2015	41 465	5380/13.0	1541/3.7	2545/6.1
	2016	43 523	5975/13.7	1760/4.0	2684/6.2
	2017	45 622	6539/14.3	1909/4.2	2699/5.9

Note

Abbreviation: GI, gastrointestinal.

a Complications with Clavien‐Dindo grades IIIa to V.

**Table 4:** Changes in the annual number of surgeries among the GI operative procedures on the esophagus

Organ	Degree of difficulty	Procedure	No. of surgeries						
			2011	2012	2013	2014	2015	2016	2017
Esophagus	Low	Cervical periesophageal abscess drainage	23	27	34	42	37	43	39
	Med	Esophageal suture (perforation, injury)	156	204	198	185	199	215	202
	Med	Thoracic periesophageal abscess drainage	22	23	18	27	27	21	21
	Med	Esophageal foreign body extraction	19	21	26	25	30	32	35
	Med	Esophageal diverticulum resection	27	32	35	48	41	34	47
	Med	Benign esophageal tumor removal	61	69	66	68	52	64	73
	Med	Esophageal resection (removal only)	388	506	580	570	571	721	720
	Med	Esophageal reconstruction: reconstruction only (gastric tube reconstruction)	699	844	888	799	848	772	828
	Med	Esophageal fistula construction	97	106	128	126	125	162	176
	Med	Esophagocardioplasty	321	418	392	398	362	365	366
	Med	Achalasia surgery	77	109	84	118	101	210	208
	High	Esophagectomy	4916	5946	5694	6091	6060	6041	6100
	High	Esophageal reconstruction: reconstruction only (colon reconstruction)	65	56	63	77	51	40	41
	High	Esophageal bypass	93	110	137	143	152	130	154
	High	Bronchoesophageal fistula surgery	6	5	9	12	7	13	5
	High	Secondary esophageal reconstruction	276	343	290	292	280	349	344

Abbreviation: GI, gastrointestinal; Med, medium.

**Table 5:** Changes in the annual number of surgeries among the GI operative procedures on the stomach and duodenum

Organ	Degree of difficulty	Procedure	No. of surgeries						
			2011	2012	2013	2014	2015	2016	2017
Stomach and duodenum	Low	Gastrostomy and suture gastrorrhaphy	52	69	74	66	65	77	64
	Low	Diverticulum, polypectomy (excluding endoscopic resection)	156	186	231	247	226	202	230
	Low	Truncal vagotomy	3	6	6	2	6	3	4
	Low	Gastroenterostomy (including duodenal jejunostomy)	4651	5330	5571	5893	5636	5633	5867
	Low	Gastric fistula construction (excluding PEG)	1717	1698	1633	1722	1790	1748	1695
	Low	Gastric pyloroplasty	116	129	115	126	100	69	82
	Low	Gastric volvulus (volvulus) surgery and rectopexy	40	38	39	0	47	42	56
	Low	Gastric suture (including gastric suture for gastric rupture, suture closure for gastroduodenal perforation, omental implantation and omental transposition)	4707	5738	5669	5837	5858	6164	5847
	Low	Local gastrectomy (including wedge resection)	2466	3108	3233	3354	3625	3766	4076
	Med	Gastrectomy (including distal gastrectomy, pylorus‐preserving gastrectomy and segmental [transverse] gastrectomy)	34 160	38 750	39 957	38 584	37 819	36 852	35 517
	Med	Selective vagotomy	8	8	10	7	6	4	6
	High	Total gastrectomy (including fundusectomy)	18 652	21 122	19 035	19 071	18 695	17 670	14 840
	High	Left upper abdominal exenteration	12	4	10	11	4	4	3

Abbreviations: GI, gastrointestinal; PEG, percutaneous endoscopic gastrostomy.

**Table 6:** Changes in the annual number of surgeries among the GI operative procedures on the small intestine and colon

Organ	Degree of difficulty	Procedure	No. of surgeries						
			2011	2012	2013	2014	2015	2016	2017
Small intestine and colon	Low	Enterotomy and enterorrhaphy	2982	3505	4025	4362	4412	4311	4378
	Low	Disinvagination (invasive)	172	250	234	239	209	242	221
	Low	Partial enterectomy (benign)	5792	7602	8564	8938	9449	9591	9465
	Low	Ileocecal resection (benign)	3238	4104	4313	4472	4523	4675	4643
	Low	Partial colectomy and sigmoid colectomy (benign)	4946	6239	6626	7358	7583	7971	8115
	Low	Appendectomy	43 437	51 316	54 421	54 319	54 897	55 168	55 261
	Low	Enterostomy and closure (without enterectomy)	15 192	19 371	21 600	23 425	24 666	25 458	26 795
	Med	Enterectomy (malignant)	2448	2703	3016	3082	3320	3360	3671
	Med	Ileocecal resection (malignant)	5492	9274	10 327	11 368	12 224	12 872	13 133
	Med	Partial colectomy and sigmoid colectomy (malignant)	25 034	29 863	31 495	32 092	33 518	33 936	32 986
	Med	Right hemicolectomy	17 890	21 034	21 814	22 446	22 850	22 829	22 543
	Med	Left hemicolectomy	5241	5347	5644	5763	6119	6178	5991
	Med	Total colectomy	2846	3131	1892	1701	1752	1735	1789
	Med	Intestinal obstruction surgery (with bowel resection)	5117	6496	7412	7775	7912	7898	24 142^a^
	Med	Enterostomy and closure (with enterectomy)	11 008	14 162	16 853	19 049	20 520	21 525	21 774
	High	Proctocolectomy and ileoanal (canal) anastomosis	308	413	441	468	499	479	452

Note

Abbreviation: GI, gastrointestinal.

a 2011‐2016: Intestinal obstruction surgery with bowel resection, 2017: Intestinal obstruction surgery with or without bowel resection.

**Table 7:** Changes in the annual number of surgeries among the GI operative procedures on the rectum and anus

Organ	Degree of difficulty	Procedure	No. of surgeries						
			2011	2012	2013	2014	2015	2016	2017
Rectum and anus	Low	Transanal rectal tumor removal	2483	3300	1657	1513	3690	3651	3761
	Low	Proctocele surgery (transanal)	1802	2461	2488	2602	2773	2805	2810
	Med	Rectectomy (benign)	300	386	2196	2060	1914	1688	1491
	Med	High anterior resection	7053	8920	8985	9496	9934	10 477	10 546
	Med	Hartmann's procedure	3562	4614	4865	5194	5650	5755	6034
	Med	Proctocele surgery (abdominoperineal)	659	996	1119	1181	1411	1538	1771
	Med	Malignant anorectal tumor excision (transanal)	1517	1037	898	864	821	778	735
	Med	Anal sphincteroplasty (by tissue replacement)	969	1378	1721	1718	2132	2045	2520
	High	Rectectomy (malignant)	5308	5828	4474	4531	4825	5096	5082
	High	Low anterior resection	16 984	20 321	21 096	21 861	22 493	21 387	20 879
	High	Pelvic evisceration	359	389	412	374	385	402	456
	High	Anorectal malignant tumor excision (posterior approach)	65	74	69	60	64	44	59

Abbreviation: GI, gastrointestinal.

**Table 8:** Changes in the annual number of surgeries among the GI operative procedures on the liver

Organ	Degree of difficulty	Procedure	No. of surgeries						
			2011	2012	2013	2014	2015	2016	2017
Liver	Low	Hepatorrhaphy	172	202	161	196	147	161	64
	Low	Liver abscess drainage (excluding percutaneous procedures)	42	47	54	44	59	55	51
	Low	Hepatic cyst resection. Suture. Drainage	425	535	606	695	695	741	861
	Low	Partial hepatectomy	9431	10 919	10 708	11 598	12 063	12 604	12 847
	Low	Liver biopsy (excluding percutaneous procedures)	122	264	176	165	175	126	138
	Low	Liver coagulonecrotic therapy (excluding percutaneous procedures)	1958	2122	1083	1069	939	854	811
	Med	Lateral segmentectomy of the liver	1390	1632	1773	1807	1666	1704	1598
	Med	Esophageal and gastric varix surgery	94	109	67	61	46	67	52
	High	Hepatectomy (segmented or more; excluding lateral segments)	7434	8239	7937	7666	7439	7610	7698
	High	Systematic subsegmentectomy	996	1353	2374	2257	2221	2367	2391
	High	Liver transplant	692	775	757	848	790	800	748
	High	Hepatopancreatoduodenectomy	99	91	118	112	138	123	138

Abbreviation: GI, gastrointestinal.

**Table 9:** Changes in the annual number of surgeries among the GI operative procedures on the gallbladder

Organ	Degree of difficulty	Procedure	No. of surgeries						
			2011	2012	2013	2014	2015	2016	2017
Gallbladder	Low	Cholangiotomy	142	163	174	139	141	132	106
	Low	Cysticolithectomy	1094	1093	750	641	611	571	63
	Low	Cholecystectomy	93 665	112 048	119 455	122 026	124 267	128 809	130 570
	Low	External cholecystostomy	104	119	127	124	109	146	143
	Low	Cystoenteric anastomosis	70	73	61	61	67	59	54
	Med	Cysticolithectomy	3682	4117	3880	3574	3342	3057	2962
	Med	Biliary tract reconstruction	150	162	265	315	362	347	332
	Med	Biliary bypass	1594	1751	1765	1686	1613	1490	1300
	Med	Cholangioplasty	201	180	192	168	156	176	128
	Med	Duodenal papilloplasty	66	68	50	33	31	37	30
	Med	Choledochal dilatation	217	240	254	242	248	291	264
	Med	Biliary fistula closure	43	42	42	37	40	34	39
	High	Malignant gallbladder tumor surgery (excluding simple cholecystectomy)	869	1013	929	963	969	948	1027
	High	Malignant bile duct tumor surgery	1268	1426	1202	1153	1155	1245	1232
	High	Biliary atresia surgery	18	18	16	20	15	18	17

Abbreviation: GI, gastrointestinal.

**Table 10:** Changes in the annual number of surgeries among the GI operative procedures on the pancreas

Organ	Degree of difficulty	Procedure	No. of surgeries						
			2011	2012	2013	2014	2015	2016	2017
Pancreas	Low	External pancreatic cyst drainage	29	27	13	21	8	13	15
	Low	External pancreatic duct drainage	17	20	26	28	22	34	13
	Med	Pancreatorrhaphy	22	17	21	34	27	17	5
	Med	Partial pancreatic resection	126	148	202	182	165	177	187
	Med	Distal pancreatectomy (benign)	1018	1398	1372	1557	1477	1536	1568
	Med	Pancreatoenteric anastomosis	81	71	59	49	44	39	35
	Med	Pancreatic (duct) anastomosis	223	295	309	388	280	269	328
	Med	Acute pancreatitis surgery	94	117	104	103	90	132	76
	Med	Pancreatolithiasis surgery	17	17	14	35	31	29	22
	Med	Plexus pancreaticus capitalis resection	1	1	2	0	1	1	0
	High	Pancreaticoduodenectomy	8305	9329	10 068	10 400	10 576	11 028	11 580
	High	Distal pancreatectomy (malignant)	2861	3344	3483	3750	3930	4173	4508
	High	Total pancreatectomy	348	408	423	496	503	545	561
	High	Duodenum‐preserving pancreas head resection	201	193	111	85	63	49	50
	High	Segmental pancreatic resection	131	163	138	165	162	169	155
	High	Distal pancreatectomy	3	2	35	20	28	27	35

Abbreviation: GI, gastrointestinal.

**Table 11:** Changes in the annual number of surgeries among the GI operative procedures on the spleen

Organ	Degree of difficulty	Procedure	No. of surgeries						
			2011	2012	2013	2014	2015	2016	2017
Spleen	Low	Splenorrhaphy	22	35	26	24	17	30	32
	Med	Splenectomy	3564	4063	4457	4215	3525	3117	2811
	Med	Partial splenic resection	23	44	26	33	26	24	21

Abbreviation: GI, gastrointestinal.

**Table 12:** Changes in the annual number of surgeries among the GI operative procedures on other organs

Organ	Degree of difficulty	Procedure	No. of surgeries						
			2011	2012	2013	2014	2015	2016	2017
Other	Low	Localized intra‐abdominal abscess surgery	2526	2944	3231	3262	2942	2764	2630
	Low	Exploratory laparotomy	5036	6852	7532	8271	8982	9629	10 416
	Med	Acute diffuse peritonitis surgery	7753	9177	10 447	12 085	13 030	13 981	14 423
	Med	Ventral hernia surgery	5053	6095	11 387	12 298	12 494	12 896	13 663
	Med	Diaphragm suture	183	218	246	213	257	253	313
	Med	Esophageal hiatus hernia surgery	511	602	725	757	800	842	981
	Med	Retroperitoneal tumor surgery	622	837	806	805	807	850	829
	Med	Abdominal wall/mesenteric/omental tumor resection	979	1398	1402	1509	1506	1707	1767
	Med	Gastrointestinal perforation closure	504	576	522	589	587	549	530
	High	Diaphragmatic hiatus hernia surgery	51	80	65	65	60	52	70

Abbreviation: GI, gastrointestinal.

### 3.2 Eight main operative procedures

The number of surgeries carried out annually for the eight main operative procedures, the percentage by gender, and the percentage according to age group between 2011 and 2017 are shown in Table 13. The percentage of patients who were ≥80 years has been increasing for all eight main procedures. Regarding the institution type in which the surgeries were carried out, more than 75% of the surgeries were carried out at certified institutions and the percentage of surgeries done at certified institutions was particularly high in 2017 for esophagectomy (95.3%), hepatectomy (non‐lateral segments; 91.2%), and pancreaticoduodenectomy (90.5%). The percentage of surgeries with participation of an anesthesiologist was more than 90% for all eight procedures. Approximately 95% of esophagectomy, hepatectomy (non‐lateral segments), and pancreaticoduodenectomy procedures involved participation of a board‐certified surgeon, whereas the percentages of right hemicolectomy and acute diffuse peritonitis surgeries with participation of a board‐certified surgeon were 76.4% and 69.0% in 2017, respectively (Table 14).

**Table 13:** Changes in the annual percentage of surgeries by gender and age group for the eight main operative procedures

Procedure	Year	No. of surgeries	Percentage by gender		Percentage according to age group (y)					
			Male	Female	<60	60 to <65	65 to <70	70 to <75	75 to <80	≥80
Esophagectomy	2011	4916	84.1	15.9	20.4	20.8	22.5	19.4	12.2	4.7
	2012	5946	84.4	15.6	19.7	21.3	20.7	20.3	13.1	4.9
	2013	5694	83.6	16.4	18.3	18.3	22.6	21.3	13.8	5.8
	2014	6091	84.0	16.0	18.7	17.8	22.8	22.0	13.4	5.2
	2015	6060	82.9	17.1	17.9	16.3	23.6	23.5	13.1	5.7
	2016	6041	81.7	18.3	17.8	15.8	25.3	21.6	14.3	5.2
	2017	6100	82.3	17.7	17.0	14.6	25.6	20.6	15.8	6.3
Gastrectomy (distal)	2011	34 160	66.6	33.4	18.1	15.0	14.2	17.4	16.8	18.5
	2012	38 750	66.9	33.1	16.9	14.8	15.0	17.8	16.5	18.8
	2013	39 957	66.7	33.3	16.3	13.5	15.8	17.8	17.6	19.0
	2014	38 584	66.4	33.6	15.7	12.4	16.6	18.4	17.3	19.5
	2015	37 819	66.6	33.4	14.8	11.3	17.5	18.2	17.5	20.6
	2016	36 852	66.6	33.4	14.5	10.4	18.5	17.6	17.4	21.6
	2017	35 517	66.8	33.2	13.4	9.9	18.0	18.1	18.0	22.6
Total gastrectomy	2011	18 652	73.7	26.3	16.6	14.7	16.0	19.7	18.0	15.0
	2012	21 122	74.2	25.8	15.5	14.8	15.7	19.2	18.5	16.3
	2013	19 035	74.0	26.0	14.7	13.5	16.9	19.4	19.2	16.3
	2014	19 071	73.7	26.3	14.0	12.3	17.2	20.1	18.9	17.5
	2015	18 695	74.5	25.5	13.7	11.1	18.9	20.8	18.2	17.4
	2016	17 670	74.4	25.6	12.6	10.3	19.6	19.5	19.0	19.0
	2017	14 840	74.2	25.8	12.2	9.9	19.0	19.6	19.8	19.5
Right hemicolectomy	2011	17 890	50.5	49.5	12.8	11.6	13.1	17.3	18.8	26.5
	2012	21 034	50.3	49.7	13.1	10.9	13.1	17.0	19.0	26.9
	2013	21 814	50.6	49.4	13.0	10.0	13.4	17.6	18.9	27.1
	2014	22 446	50.6	49.4	12.0	9.2	13.8	18.2	18.6	28.2
	2015	22 850	50.5	49.5	11.5	8.6	14.6	18.1	18.1	29.1
	2016	22 829	51.3	48.7	11.4	7.7	15.9	16.7	18.5	29.8
	2017	22 543	50.9	49.1	11.3	7.4	14.9	16.3	19.3	30.8
Low anterior resection	2011	16 984	64.8	35.2	24.1	18.5	16.5	16.2	12.9	11.7
	2012	20 321	64.8	35.2	24.2	17.6	16.5	16.6	13.1	12.0
	2013	21 096	64.2	35.8	23.8	16.5	17.4	16.9	13.5	11.8
	2014	21 861	64.8	35.2	23.1	15.7	18.3	17.9	13.1	11.9
	2015	22 493	64.4	35.6	23.5	14.2	19.6	17.1	13.6	12.0
	2016	21 387	64.4	35.6	23.4	13.6	20.7	16.8	13.2	12.2
	2017	20 879	64.2	35.8	23.2	12.6	20.9	16.7	13.5	13.2
Hepatectomy (non‐lateral segments)	2011	7434	70.4	29.6	20.1	16.4	16.5	20.4	18.0	8.7
	2012	8239	69.5	30.5	19.8	16.1	17.4	19.5	18.5	8.8
	2013	7937	69.4	30.6	19.4	14.2	18.0	20.3	18.2	9.9
	2014	7666	69.2	30.8	18.5	13.8	18.5	21.5	17.6	10.0
	2015	7439	68.9	31.1	18.7	12.5	19.3	20.9	17.6	11.1
	2016	7610	68.7	31.3	18.0	11.9	21.1	20.4	17.5	11.1
	2017	7698	69.5	30.5	17.2	11.3	20.5	20.4	18.7	11.9
Pancreaticoduodenectomy	2011	8305	61.9	38.1	16.1	16.0	17.3	20.9	18.8	10.9
	2012	9329	62.0	38.0	14.7	15.8	18.0	20.6	20.2	10.6
	2013	10 068	60.9	39.1	14.0	12.6	19.6	22.5	19.4	11.8
	2014	10 400	59.5	40.5	18.4	12.4	19.0	21.0	18.2	11.1
	2015	10 576	60.7	39.3	14.2	11.7	20.0	22.9	19.3	12.0
	2016	11 028	61.1	38.9	14.2	10.3	20.6	21.8	20.3	12.7
	2017	11 580	61.1	38.9	13.8	9.8	20.4	20.8	21.6	13.6
Acute diffuse peritonitis surgery	2011	7753	60.0	40.0	31.4	11.2	9.7	11.7	13.2	22.9
	2012	9177	61.0	39.0	30.3	11.2	10.1	11.6	13.4	23.4
	2013	10 447	60.1	39.9	29.1	10.3	11.5	11.8	13.1	24.1
	2014	12 085	61.2	38.8	28.4	9.5	12.2	12.3	12.9	24.7
	2015	13 030	59.4	40.6	28.2	8.9	12.5	13.1	12.3	25.0
	2016	13 981	60.2	39.8	27.4	8.6	13.4	12.4	12.3	26.0
	2017	14 423	59.4	40.6	26.5	7.8	13.0	12.0	13.6	27.1

**Table 14:** Institution type, anesthesiologist and specialist participation rates in the eight main operative procedures

Procedure	Year	No. of surgeries	Percentage by institution type			Anesthesiologist participation (%)	Board‐certified surgeon participation (%)	Medical practitioners (%)	
			Certified institution	Related institution	Other			Board‐certified surgeons	Non‐board‐certified surgeons
Esophagectomy	2011	4916	94.2	5.3	0.5	97.6	88.4	63.5	36.5
	2012	5946	78.3	4.9	16.8	98.1	89.0	64.8	35.2
	2013	5694	92.9	5.9	1.2	98.0	90.8	66.6	33.4
	2014	6091	93.6	4.7	1.7	98.6	92.6	70.2	29.8
	2015	6060	93.6	4.6	1.8	98.5	93.5	72.1	27.9
	2016	6041	94.5	3.8	1.7	98.8	93.7	73.2	26.8
	2017	6100	95.3	3.1	1.7	98.8	94.8	74.7	25.3
Gastrectomy (distal)	2011	34 160	81.1	16.6	2.3	93.2	71.3	37.0	63.0
	2012	38 750	64.5	15.2	20.3	93.9	72.5	37.9	62.1
	2013	39 957	76.6	19.2	4.1	93.6	76.1	40.6	59.4
	2014	38 584	77.7	17.8	4.5	94.0	78.4	42.1	57.9
	2015	37 819	77.3	18.3	4.4	94.1	78.1	41.3	58.7
	2016	36 852	80.2	15.9	4.0	95.0	81.8	43.8	56.2
	2017	35 517	80.2	14.9	4.8	95.4	82.4	45.2	54.8
Total gastrectomy	2011	18 652	80.9	16.8	2.3	93.9	71.6	37.4	62.6
	2012	21 122	63.0	15.3	21.7	94.3	72.1	38.0	62.0
	2013	19 035	77.2	18.9	3.9	94.2	75.0	39.5	60.5
	2014	19 071	77.8	17.9	4.3	94.4	77.7	41.7	58.3
	2015	18 695	77.9	17.9	4.1	94.5	78.2	42.6	57.4
	2016	17 670	80.0	15.9	4.0	95.0	81.4	45.0	55.0
	2017	14 840	79.3	15.8	4.9	95.0	80.7	44.3	55.7
Right hemicolectomy	2011	17 890	75.7	21.2	3.1	92.7	66.0	30.5	69.5
	2012	21 034	60.0	18.3	21.7	93.0	67.1	30.8	69.2
	2013	21 814	72.1	22.3	5.6	92.9	69.7	32.6	67.4
	2014	22 446	71.2	23.1	5.7	93.4	71.9	33.6	66.4
	2015	22 850	72.1	22.0	5.9	94.1	72.4	33.5	66.5
	2016	22 829	73.8	20.1	6.1	94.5	74.2	34.3	65.7
	2017	22 543	75.0	18.4	6.6	94.7	76.4	37.1	62.9
Low anterior resection	2011	16 984	79.4	17.7	2.9	93.4	72.7	41.6	58.4
	2012	20 321	64.0	16.2	19.7	93.8	73.0	42.3	57.7
	2013	21 096	76.3	19.5	4.2	93.7	75.5	44.3	55.7
	2014	21 861	76.2	19.0	4.9	94.4	78.2	47.2	52.8
	2015	22 493	76.9	18.3	4.8	94.6	79.2	47.7	52.3
	2016	21 387	79.0	16.4	4.7	95.0	81.0	48.8	51.2
	2017	20 879	79.3	15.6	5.1	95.2	83.1	51.2	48.8
Hepatectomy (non‐lateral segments)	2011	7434	91.1	8.0	0.8	96.4	88.9	61.5	38.5
	2012	8239	75.9	7.9	16.3	96.8	89.3	64.0	36.0
	2013	7937	88.1	9.7	2.2	96.9	91.0	65.2	34.8
	2014	7666	88.2	8.7	3.1	96.7	92.3	66.6	33.4
	2015	7439	89.2	8.6	2.2	97.2	92.3	66.6	33.4
	2016	7610	90.7	7.1	2.1	97.1	93.3	67.7	32.3
	2017	7698	91.2	6.6	2.2	97.7	95.1	72.3	27.7
Pancreaticoduodenectomy	2011	8305	87.8	11.0	1.2	95.9	85.7	58.7	41.3
	2012	9329	72.4	8.8	18.8	96.6	87.2	60.9	39.1
	2013	10 068	85.9	11.7	2.4	96.0	87.9	60.5	39.5
	2014	10 400	86.4	10.4	3.3	96.4	90.3	62.2	37.8
	2015	10 576	88.5	9.2	2.4	96.9	90.9	62.1	37.9
	2016	11 028	89.4	8.3	2.3	97.1	91.7	63.3	36.7
	2017	11 580	90.5	7.2	2.3	97.3	93.0	65.0	35.0
Acute diffuse peritonitis surgery	2011	7753	80.6	16.9	2.4	90.0	58.5	23.5	76.5
	2012	9177	65.2	16.4	18.4	90.4	59.4	22.7	77.3
	2013	10 447	77.7	18.1	4.2	91.2	62.4	23.9	76.1
	2014	12 085	77.7	17.2	5.1	91.9	63.3	25.1	74.9
	2015	13 030	79.8	15.9	4.3	92.2	64.5	24.9	75.1
	2016	13 981	82.2	13.8	4.0	93.0	66.8	26.1	73.9
	2017	14 423	83.1	13.0	3.8	93.3	69.0	27.2	72.8

The rate of preoperative chemotherapy within 90 days increased over time and was 50.9% for esophagectomy in 2017. Although the rates of preoperative chemotherapy in patients who underwent total gastrectomy, low anterior resection, or pancreaticoduodenectomy were relatively low (<10%), the rates of preoperative chemotherapy in patients who underwent these procedures showed an increasing trend over time (Table 15).

**Table 15:** Changes in the annual number of surgeries in patients who received preoperative chemotherapy or radiation prior to the eight main operative procedures

	Year	No. of surgeries	No. with preoperative chemotherapy within 30 d/rate (%)	No. with preoperative chemotherapy within 90 d/rate (%)	No. with preoperative radiotherapy within 90 d/rate (%)
Esophagectomy	2011	4914	928/18.9	–/–	235/4.8
	2012	5947	1131/19.0	2476/41.6	432/7.3
	2013	5694	982/17.2	2386/41.9	374/6.6
	2014	6092	1145/18.8	2733/44.9	435/7.1
	2015	6058	1153/19.0	2842/46.9	416/6.9
	2016	6041	1150/19.0	2955/48.9	398/6.6
	2017	6100	1103/18.1	3103/50.9	400/6.6
Gastrectomy (distal)	2011	32 241	469/1.5	–/–	47/0.1
	2012	36 715	502/1.4	902/2.5	45/0.1
	2013	39 094	516/1.3	1028/2.6	51/0.1
	2014	37 718	479/1.3	1002/2.7	36/0.1
	2015	37 082	492/1.3	990/2.7	50/0.1
	2016	36 197	481/1.3	1070/3.0	36/0.1
	2017	34 861	462/1.3	1073/3.1	46/0.1
Total gastrectomy	2011	18 046	814/4.5	–/–	33/0.2
	2012	20 467	835/4.1	1540/7.5	31/0.2
	2013	18 777	656/3.5	1364/7.3	48/0.3
	2014	17 962	713/4.0	1002/5.6	48/0.3
	2015	17 385	638/3.7	1452/8.4	38/0.2
	2016	16 188	583/3.6	1366/8.4	34/0.2
	2017	14 840	566/3.8	1385/9.3	28/0.2
Right hemicolectomy	2011	17 884	157/0.9	–/–	24/0.1
	2012	21 027	184/0.9	317/1.5	32/0.2
	2013	21 816	187/0.9	363/1.7	0.1
	2014	22 444	192/0.9	370/1.6	29/0.1
	2015	22 851	204/0.9	409/1.8	53/0.2
	2016	22 829	256/1.1	438/1.9	56/0.2
	2017	22 543	192/0.9	416/1.8	46/0.2
Low anterior resection	2011	16 982	355/2.1	–/–	293/1.7
	2012	20 319	481/2.4	1131/5.6	484/2.4
	2013	21 097	477/2.3	1273/6.0	523/2.5
	2014	21 854	531/2.4	1533/7.0	641/2.9
	2015	22 496	565/2.5	1721/7.7	599/2.7
	2016	21 387	507/2.4	1682/7.9	627/2.9
	2017	20 879	526/2.5	1759/8.4	665/3.2
Hepatectomy (non‐lateral segments)	2011	7439	420/5.6	–/–	31/0.4
	2012	8242	508/6.2	1290/15.7	38/0.5
	2013	7937	454/5.7	1293/16.3	36/0.5
	2014	7663	419/5.5	1170/15.3	32/0.4
	2015	7439	358/4.8	1152/15.5	25/0.3
	2016	7610	350/4.6	1121/14.7	46/0.6
	2017	7698	326/4.2	1137/14.8	64/0.8
Pancreaticoduodenectomy	2011	8306	227/2.7	–/–	88/1.1
	2012	9336	229/2.5	440/4.7	155/1.7
	2013	10 069	291/2.9	584/5.8	213/2.1
	2014	10 395	304/2.9	631/6.1	214/2.1
	2015	10 577	339/3.2	766/7.2	272/2.6
	2016	11 028	374/3.4	850/7.7	268/2.4
	2017	11 580	410/3.5	907/7.8	240/2.1
Acute diffuse peritonitis surgery	2011	7751	277/3.6	–/–	47/0.6
	2012	9182	352/3.8	463/5.0	61/0.7
	2013	10 452	412/3.9	573/5.5	62/0.6
	2014	12 085	396/3.3	570/4.7	60/0.5
	2015	13 030	483/3.7	669/5.1	76/0.6
	2016	13 981	511/3.7	732/5.2	88/0.6
	2017	14 423	553/3.8	762/5.3	82/0.6

‐/‐ indicates lack of data

The number and rate of comorbidities and American Society of Anesthesiologists (ASA) score in patients who underwent the eight main procedures are shown in Table 16. The annual rates of diabetes mellitus and hypertension increased over time for all eight procedures.

**Table 16:** Changes in the annual number of surgeries in patients with preoperative comorbidities among patients who underwent the eight main operative procedures

	Year	No. of surgeries	No. with diabetes mellitus/rate (%)	No. with dyspnea within 30 d/rate (%)	No. with COPD/rate (%)	No. with hypertension within 30 d/rate (%)	No. with myocardial infarction within 6 mo/rate (%)	No. receiving dialysis within 14 d/rate (%)	Percentage according to ASA score (%)				
									1	2	3	4	5
Esophagectomy	2011	4914	628/12.8	102/2.1	302/6.1	1531/31.2	8/0.2	13/0.3	39.5	52.9	7.4	0.1	0.0
	2012	5947	773/13.0	92/1.5	374/6.3	1881/31.6	22/0.4	12/0.2	31.8	61.0	6.8	0.2	0.2
	2013	5694	737/12.9	98/1.7	360/6.3	1944/34.1	17/0.3	16/0.3	29.5	62.5	7.7	0.2	0.1
	2014	6092	824/13.5	100/1.6	497/8.2	2144/35.2	17/0.3	8/0.1	27.6	64.7	7.5	0.2	0.1
	2015	6058	895/14.8	82/1.4	527/8.7	2200/36.3	13/0.2	22/0.4	24.3	67.6	7.8	0.2	0.1
	2016	6041	895/14.8	76/1.3	501/8.3	2233/37.0	12/0.2	23/0.4	19.7	71.7	8.4	0.1	0.1
	2017	6100	941/15.4	72/1.2	469/7.7	2357/38.6	11/0.2	20/0.3	16.5	72.4	10.9	0.1	0.1
Gastrectomy (distal)	2011	32 250	5079/15.7	737/2.3	1182/3.7	11 192/34.7	185/0.6	271/0.8	40.5	49.7	9.2	0.4	0.2
	2012	36 689	6143/16.7	772/2.1	1316/3.6	13 397/36.5	249/0.7	294/0.8	35.2	54.0	10.1	0.5	0.2
	2013	39 094	6742/17.2	740/1.9	1639/4.2	14 665/37.5	211/0.5	331/0.8	31.7	57.1	10.6	0.4	0.2
	2014	37 719	6652/17.6	702/1.9	1754/4.7	14 677/38.9	161/0.4	290/0.8	28.7	59.8	11.0	0.4	0.1
	2015	37 083	6830/18.4	649/1.8	1833/4.9	15 023/40.5	174/0.5	279/0.8	25.7	62.2	11.7	0.4	0.1
	2016	36 197	6791/18.8	627/1.7	1890/5.2	14 910/41.2	175/0.5	295/0.8	23.1	63.9	12.4	0.4	0.1
	2017	34 862	6580/18.9	563/1.6	1693/4.9	14 631/42.0	159/0.5	283/0.8	19.9	66.4	13.2	0.4	0.1
Total gastrectomy	2011	18 048	2951/16.4	448/2.5	796/4.4	5983/33.2	125/0.7	95/0.5	38.0	52.2	9.2	0.4	0.2
	2012	20 462	3424/16.7	482/2.4	868/4.2	7335/35.8	166/0.8	125/0.6	33.0	56.2	10.0	0.5	0.3
	2013	18 775	3304/17.6	368/2.0	880/4.7	6999/37.3	98/0.5	116/0.6	28.8	59.8	10.8	0.4	0.2
	2014	17 963	3332/18.5	346/1.9	922/5.1	6850/38.1	81/0.5	105/0.6	25.6	62.4	11.4	0.4	0.1
	2015	17 387	3178/18.3	341/2.0	915/5.3	6761/38.9	65/0.4	109/0.6	23.3	64.5	11.6	0.4	0.2
	2016	16 191	3117/19.3	322/2.0	917/5.7	6535/40.4	79/0.5	94/0.6	20.8	65.8	12.8	0.5	0.1
	2017	14 840	2875/19.4	271/1.8	784/5.3	6046/40.7	71/0.5	98/0.7	17.4	68.7	13.2	0.5	0.1
Right hemicolectomy	2011	17 885	3073/17.2	518/2.9	526/2.9	6495/36.3	125/0.7	182/1.0	31.8	53.8	13.0	1.0	0.4
	2012	21 022	3564/17.0	573/2.7	586/2.8	7830/37.2	155/0.7	210/1.0	29.2	56.0	13.3	1.1	0.4
	2013	21 816	3802/17.4	470/2.2	614/2.8	8431/38.6	112/0.5	223/1.0	26.4	58.3	13.8	1.0	0.4
	2014	22 444	4230/18.8	514/2.3	684/3.0	9048/40.3	118/0.5	226/1.0	23.1	61.3	14.4	0.9	0.3
	2015	22 851	4355/19.1	471/2.1	705/3.1	9419/41.2	121/0.5	216/0.9	21.0	62.3	15.5	0.9	0.4
	2016	22 829	4484/19.6	477/2.1	721/3.2	9575/41.9	88/0.4	247/1.1	19.0	63.3	16.4	1.0	0.3
	2017	22 543	4481/19.9	447/2.0	715/3.2	9535/42.3	105/0.5	274/1.2	16.4	64.7	17.4	1.1	0.4
Low anterior resection	2011	16 981	2908/17.1	259/1.5	443/2.6	5321/31.3	72/0.4	80/0.5	42.1	50.3	7.5	0.1	0.0
	2012	20 306	3421/16.8	300/1.5	524/2.6	6533/32.2	102/0.5	102/0.5	38.7	52.7	8.3	0.2	0.1
	2013	21 097	3505/16.6	241/1.1	597/2.8	6965/33.0	75/0.4	112/0.5	35.5	55.7	8.4	0.3	0.1
	2014	21 854	3836/17.6	277/1.3	851/3.9	7634/34.9	90/0.4	105/0.5	32.1	58.7	8.9	0.2	0.1
	2015	22 496	4013/17.8	263/1.2	755/3.4	7917/35.2	101/0.4	116/0.5	29.9	60.8	8.9	0.2	0.1
	2016	21 387	3855/18.0	221/1.0	797/3.7	7693/36.0	68/0.3	104/0.5	27.5	62.5	9.7	0.2	0.1
	2017	20 879	3885/18.6	236/1.1	720/3.4	7512/36.0	67/0.3	108/0.5	24.2	65.4	10.1	0.3	0.1
Hepatectomy (non‐lateral segments)	2011	7439	1852/24.9	125/1.7	202/2.7	2728/36.7	39/0.5	60/0.8	33.9	55.7	9.9	0.3	0.1
	2012	8242	2061/25.0	100/1.2	235/2.9	3112/37.8	36/0.4	63/0.8	28.1	61.0	10.4	0.3	0.2
	2013	7937	1975/24.9	79/1.0	253/3.2	3155/39.8	28/0.4	71/0.9	23.4	65.0	11.2	0.2	0.1
	2014	7663	1968/25.7	100/1.3	290/3.8	3066/40.0	28/0.4	55/0.7	20.2	68.3	11.0	0.4	0.1
	2015	7439	1973/26.5	90/1.2	299/4.0	3059/41.1	27/0.4	71/1.0	17.9	69.3	12.3	0.4	0.1
	2016	7610	2026/26.6	78/1.0	293/3.9	3269/43.0	16/0.2	66/0.9	15.6	72.6	11.4	0.3	0.1
	2017	7698	2153/28.0	79/1.0	308/4.0	3418/44.4	20/0.3	67/0.9	13.7	73.2	12.9	0.1	0.1
Pancreaticoduodenectomy	2011	8306	2280/27.5	95/1.1	227/2.7	2819/33.9	36/0.4	50/0.6	34.3	56.5	8.9	0.2	0.2
	2012	9331	2660/28.5	113/1.2	247/2.6	3297/35.3	38/0.4	49/0.5	28.1	62.3	9.2	0.3	0.1
	2013	10 069	2830/28.1	84/0.8	269/2.7	3729/37.0	45/0.4	60/0.6	24.9	64.4	10.4	0.2	0.1
	2014	10 395	3011/29.0	88/0.8	362/3.5	3973/38.2	34/0.3	58/0.6	22.6	66.2	10.9	0.2	0.0
	2015	10 577	3057/28.9	92/0.9	385/3.6	4150/39.2	36/0.3	48/0.5	19.1	69.3	11.3	0.2	0.1
	2016	11 028	3321/30.1	112/1.0	443/4.0	4380/39.7	31/0.3	62/0.6	16.7	70.3	12.6	0.2	0.1
	2017	11 580	3517/30.4	97/0.8	416/3.6	4712/40.7	36/0.3	66/0.6	14.6	72.8	12.4	0.1	0.1
Acute diffuse peritonitis surgery	2011	7751	1063/13.7	655/8.5	264/3.4	2252/29.1	49/0.6	306/3.9	20.7	35.3	29.0	9.9	5.1
	2012	9179	1253/13.7	737/8.0	300/3.3	2799/30.5	70/0.8	341/3.7	19.3	36.6	30.2	9.6	4.3
	2013	10 452	1422/13.6	731/7.0	348/3.3	3306/31.6	63/0.6	411/3.9	17.3	36.5	33.7	8.8	3.7
	2014	12 085	1745/14.4	758/6.3	416/3.4	3913/32.4	65/0.5	444/3.7	16.0	37.6	34.4	9.2	2.8
	2015	13 030	1862/14.3	732/5.6	366/2.8	4338/33.3	62/0.5	466/3.6	13.9	40.4	33.9	9.0	2.8
	2016	13 981	2113/15.1	765/5.5	441/3.2	4811/34.4	79/0.6	517/3.7	13.2	40.0	34.5	9.7	2.7
	2017	14 423	2259/15.7	724/5.0	386/2.7	5118/35.5	63/0.4	496/3.4	11.4	40.6	36.3	9.2	2.5

Abbreviations: ASA, American Society of Anesthesiologists; COPD, chronic obstructive pulmonary disease.

**Figure 2:**
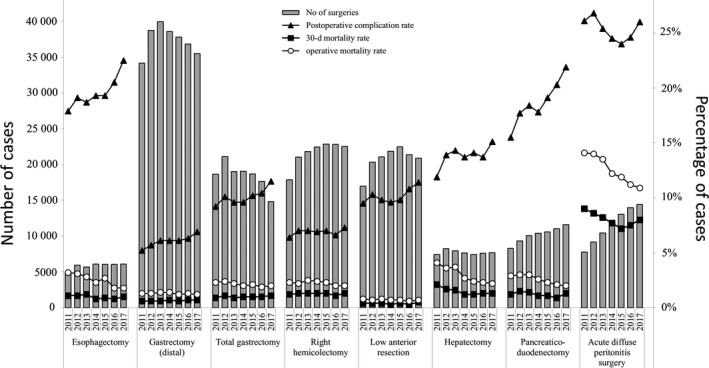
Annual changes in the number of surgeries, postoperative complication rate, operative mortality rate, and 30‐d postoperative mortality rate: analysis of the eight major surgical procedures. Postoperative complication rate: the rate of complications with Clavien‐Dindo (C‐D) classification of grade III or higher

Table 17 and Figure 2 show the morbidity and mortality rates of the eight main operative procedures. Other than for acute diffuse peritonitis surgery, the operative mortality rates for the procedures were 0.6%‐4.1%, and the postoperative 30‐day mortality rates were 0.3%‐2.1%. The operative mortality rate and the 30‐day postoperative mortality rate for acute diffuse peritonitis surgery were 10.9% and 8.0% in 2017, respectively. The annual numbers of cases of gastrectomy and total gastrectomy have been decreasing, and those of pancreaticoduodenectomy and acute diffuse peritonitis surgery have been increasing over time. Although there were differences in the incidences of complications and mortality according to the procedure, the annual postoperative complication rate of the eight main procedures generally increased and operative mortality rate generally decreased over time.

**Table 17:** Annual number of surgeries and mortality rates among patients who underwent the eight main operative procedures

Procedure	Year	No. of surgeries	No. of postoperative complications^a^/rate (%)	No. of reoperations/rate (%)	No. of postoperative	No. of postoperative
					30‐d mortalities/rate (%)	90‐d mortalities/rate (%)
Esophagectomy	2011	4916	879/17.9	310/6.3	55/1.1	158/3.2
	2012	5946	1135/19.1	345/5.8	63/1.1	183/3.1
	2013	5694	1067/18.7	375/6.6	67/1.2	161/2.8
	2014	6091	1178/19.3	367/6.0	49/0.8	140/2.3
	2015	6060	1171/19.3	392/6.5	57/0.9	166/2.7
	2016	6041	1240/20.5	357/5.9	49/0.8	109/1.8
	2017	6100	1374/22.5	355/5.8	61/1.0	108/1.8
Gastrectomy (distal)	2011	34 160	1774/5.2	709/2.1	208/0.6	451/1.3
	2012	38 750	2205/5.7	849/2.2	232/0.6	516/1.3
	2013	39 957	2450/6.1	892/2.2	239/0.6	542/1.4
	2014	38 584	2356/6.1	941/2.4	264/0.7	523/1.4
	2015	37 819	2325/6.1	851/2.3	222/0.6	452/1.2
	2016	36 852	2314/6.3	825/2.2	249/0.7	473/1.3
	2017	35 517	2445/6.9	859/2.4	253/0.7	437/1.2
Total gastrectomy	2011	18 652	1716/9.2	634/3.4	177/0.9	427/2.3
	2012	21 122	2135/10.1	758/3.6	224/1.1	503/2.4
	2013	19 035	1831/9.6	642/3.4	169/0.9	428/2.2
	2014	19 071	1840/9.6	698/3.7	185/1.0	379/2.0
	2015	18 695	1907/10.2	654/3.5	178/1.0	387/2.1
	2016	17 670	1835/10.4	638/3.6	174/1.0	336/1.9
	2017	14 840	1702/11.5	514/3.5	161/1.1	293/2.0
Right hemicolectomy	2011	17 890	1150/6.4	588/3.3	213/1.2	410/2.3
	2012	21 034	1470/7.0	677/3.2	263/1.3	471/2.2
	2013	21 814	1527/7.0	721/3.3	280/1.3	538/2.5
	2014	22 446	1544/6.9	771/3.4	287/1.3	530/2.4
	2015	22 850	1607/7.0	769/3.4	301/1.3	534/2.3
	2016	22 829	1510/6.6	791/3.5	253/1.1	449/2.0
	2017	22 543	1648/7.3	785/3.5	296/1.3	450/2.0
Low anterior resection	2011	16 984	1616/9.5	1213/7.1	75/0.4	136/0.8
	2012	20 321	2092/10.3	1413/6.9	88/0.4	149/0.7
	2013	21 096	2059/9.8	1473/7.0	80/0.4	175/0.8
	2014	21 861	2098/9.6	1546/7.1	70/0.3	152/0.7
	2015	22 493	2210/9.8	1550/6.9	95/0.4	156/0.7
	2016	21 387	2306/10.8	1492/7.0	68/0.3	126/0.6
	2017	20 879	2376/11.4	1330/6.4	96/0.5	148/0.7
Hepatectomy (non‐lateral segments)	2011	7434	886/11.9	203/2.7	155/2.1	303/4.1
	2012	8239	1146/13.9	248/3.0	142/1.7	293/3.6
	2013	7937	1135/14.3	226/2.8	130/1.6	290/3.7
	2014	7666	1052/13.7	242/3.2	94/1.2	208/2.7
	2015	7439	1049/14.1	213/2.9	87/1.2	182/2.4
	2016	7610	1046/13.7	220/2.9	96/1.3	178/2.3
	2017	7698	1160/15.1	221/2.9	97/1.3	169/2.2
Pancreaticoduodenectomy	2011	8305	1285/15.5	299/3.6	97/1.2	238/2.9
	2012	9329	1654/17.7	365/3.9	137/1.5	281/3.0
	2013	10 068	1853/18.4	407/4.0	142/1.4	307/3.0
	2014	10 400	1847/17.8	374/3.6	111/1.1	267/2.6
	2015	10 576	2025/19.1	378/3.6	120/1.1	247/2.3
	2016	11 028	2242/20.3	393/3.6	98/0.9	232/2.1
	2017	11 580	2539/21.9	413/3.6	145/1.3	232/2.0
Acute diffuse peritonitis surgery	2011	7753	2022/26.1	634/8.2	697/9.0	1096/14.1
	2012	9177	2456/26.8	685/7.5	785/8.6	1289/14.0
	2013	10 447	2652/25.4	786/7.5	861/8.2	1408/13.5
	2014	12 085	2966/24.5	937/7.8	927/7.7	1472/12.2
	2015	13 030	3126/24.0	1051/8.1	943/7.2	1551/11.9
	2016	13 981	3445/24.6	1068/7.6	1052/7.5	1572/11.2
	2017	14 423	3756/26.0	1125/7.8	1152/8.0	1575/10.9

a Complications with Clavien‐Dindo grades IIIa to V are shown.

**Figure 3:**
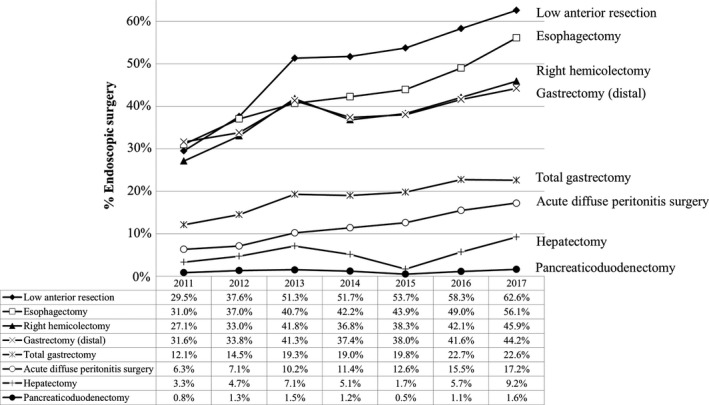
Annual changes in the percentage of surgeries carried out by endoscopic surgery: analysis of the eight major surgical procedures

The increase in the incidence of endoscopic surgery over time is shown in Figure 3 and Table 18. The annual percentage of surgeries carried out by endoscopic surgery is greatly increasing in low anterior resection and esophagectomy over time. In contrast, laparoscopic hepatectomy and pancreaticoduodenectomy have been carried out in a limited number of institutions.

**Table 18:** Changes in the annual percentage of surgeries carried out by endoscopic surgery for the eight main operative procedures

Procedure	Year	No. of surgeries	Endoscopic surgery	% Endoscopic surgery
Esophagectomy	2011	4917	1525	31.0
	2012	5948	2200	37.0
	2013	5694	2315	40.7
	2014	6091	2569	42.2
	2015	6060	2659	43.9
	2016	6041	2961	49.0
	2017	6100	3424	56.1
Gastrectomy (distal)	2011	34 198	10 801	31.6
	2012	38 774	13 098	33.8
	2013	39 959	16 507	41.3
	2014	38 584	14 432	37.4
	2015	37 819	14 357	38.0
	2016	36 852	15 333	41.6
	2017	35 517	15 696	44.2
Total gastrectomy	2011	18 674	2258	12.1
	2012	21 139	3060	14.5
	2013	19 038	3669	19.3
	2014	19 071	3620	19.0
	2015	18 695	3707	19.8
	2016	17 670	4007	22.7
	2017	14 840	3347	22.6
Right hemicolectomy	2011	17 899	4842	27.1
	2012	21 047	6954	33.0
	2013	21 816	9124	41.8
	2014	22 446	8269	36.8
	2015	22 850	8755	38.3
	2016	22 829	9622	42.1
	2017	22 543	10 341	45.9
Low anterior resection	2011	16 996	5018	29.5
	2012	20 333	7649	37.6
	2013	21 098	10 814	51.3
	2014	21 861	11 298	51.7
	2015	22 493	12 080	53.7
	2016	21 387	12 478	58.3
	2017	20 879	13 064	62.6
Hepatectomy (non‐lateral segments)	2011	7440	242	3.3
	2012	8246	389	4.7
	2013	7938	567	7.1
	2014	7666	392	5.1
	2015	7439	127	1.7
	2016	7610	433	5.7
	2017	7698	712	9.2
Pancreaticoduodenectomy	2011	8310	67	0.8
	2012	9340	121	1.3
	2013	10 069	156	1.5
	2014	10 400	124	1.2
	2015	10 576	53	0.5
	2016	11 028	118	1.1
	2017	11 580	188	1.6
Acute diffuse peritonitis surgery	2011	7767	488	6.3
	2012	9189	652	7.1
	2013	10 452	1070	10.2
	2014	12 085	1381	11.4
	2015	13 030	1638	12.6
	2016	13 981	2164	15.5
	2017	14 423	2478	17.2

## 4 DISCUSSION

Since the start of NCD registration in 2011, a robust nationwide database has been constructed as a result of the work of data managers and surgeons at the participating hospitals. We can see the real clinical status of surgical outcomes in Japan. The gastroenterological section of the NCD database shows three features: aging of the population, low mortality rate, and increase in endoscopic surgery.

The Japanese Ministry of Internal Affairs and Communications reported that the percentage of senior citizens aged 65 years or over among the Japanese population was 27.7% and the percentage of those aged 75 years or over was 13.8% in 2017, and these are the highest percentages in the world.^21^ It has been estimated that aging of the population will progress and the percentage aged ≥65 years will increase to a little less than 40% in 2050. Our data showed that 50% of right hemicolectomies and 40% of gastrectomies were carried out in patients aged 75 years or over in 2017. With the increase in the aging population, the annual rates of preoperative comorbidities such as diabetes mellitus and hypertension also increased. Age category was reported as a risk factor for operative mortality in all eight main procedures.^2^

In spite of the high population of aged patients, the mortality rates for all of the procedures seemed to be acceptable as a nationwide outcome, as they are satisfactorily lower than those reported from other countries.^22^, ^23^ These results may be explained by the high participation rate of board‐certified surgeons in gastroenterological surgeries (BCS‐Gs). The association between the participation of BCS‐Gs and mortality was evaluated using 250 012 surgical cases registered in 2011 and 2012. The participation of BCS‐Gs contributed to favorable outcomes especially for distal gastrectomy and pancreaticoduodenectomy.^19^ From 2011 to 2017, the annual percentage of surgeries with participation of a board‐certified surgeon in the eight procedures gradually increased and the operative mortality was kept at a low level. Centralization of the surgical center may also be important for improving surgical outcomes. The operative mortality rate after distal gastrectomy definitively decreased as both surgeon volume and hospital volume increased.^24^ After risk adjustment for surgeon and hospital volume and patient characteristics, hospital volume (≥52 cases per year) was significantly associated with low operative mortality.^24^ As for esophagectomy, high‐volume hospitals (≥30 cases per year) had a lower risk‐adjusted mortality rate compared with low‐volume hospitals (≤10 cases).^25^

Although the postoperative mortality was kept at a low level, the annual rate of postoperative complications with C‐D classification of grade III or higher gradually increased over time. The reason for this increase in complications may be related to the aging population with increased comorbidities. The factors causing these phenomena should be investigated for each procedure. To improve short‐term surgical outcomes in aged patients, minimally invasive surgery might play a pivotal role. The annual rate of endoscopic surgery dramatically increased from 2011 to 2017. Studies using the NCD data showed that the length of hospital stay was significantly shorter in patients who underwent endoscopic surgery.^26^, ^27^

The NCD is currently estimated to contain data on approximately 95% of all surgical cases in Japan.^28^ The NCD provides transparency of surgical outcomes.^2^ Using the NCD, many studies have been conducted and other studies are in progress to improve surgical outcomes.

## DISCLOSURE

Conflicts of Interest: Authors declare no conflicts of interest for this article.
